# Optical Addressing of Multi-Colour Photochromic Material Mixture for Volumetric Display

**DOI:** 10.1038/srep31543

**Published:** 2016-08-16

**Authors:** Ryuji Hirayama, Atsushi Shiraki, Makoto Naruse, Shinichiro Nakamura, Hirotaka Nakayama, Takashi Kakue, Tomoyoshi Shimobaba, Tomoyoshi Ito

**Affiliations:** 1 Graduate School of Engineering, Chiba University, 1-33 Yayoi-cho, Inage-ku, Chiba 263-8522, Japan; 2 Research Fellow of the Japan Society for the Promotion of Science, 5-3-1 Kojimachi, Chiyoda-ku, Tokyo 102-0083, Japan; 3 Institute of Management and Information Technologies, Chiba University, 1-33 Yayoi-cho, Inage-ku, Chiba 263-8522, Japan; 4 Network System Research Institute, National Institute of Information and Communications Technology, 4-2-1 Nukui-kita, Koganei, Tokyo 184-8795, Japan; 5 Innovation Center, RIKEN, 2-1 Hirosawa, Wako, Saitama 351-0198, Japan; 6 Center for Computational Astrophysics, National Astronomical Observatory of Japan, 2-21-1 Osawa, Mitaka, Tokyo 181-8588, Japan

## Abstract

This is the first study to demonstrate that colour transformations in the volume of a photochromic material (PM) are induced at the intersections of two control light channels, one controlling PM colouration and the other controlling decolouration. Thus, PM colouration is induced by position selectivity, and therefore, a dynamic volumetric display may be realised using these two control lights. Moreover, a *mixture* of multiple PM types with different absorption properties exhibits different colours depending on the control light spectrum. Particularly, the spectrum management of the control light allows colour-selective colouration besides position selectivity. Therefore, a PM-based, full-colour volumetric display is realised. We experimentally construct a mixture of two PM types and validate the operating principles of such a volumetric display system. Our system is constructed simply by mixing multiple PM types; therefore, the display hardware structure is extremely simple, and the minimum size of a volume element can be as small as the size of a molecule. Volumetric displays can provide natural three-dimensional (3D) perception; therefore, the potential uses of our system include high-definition 3D visualisation for medical applications, architectural design, human–computer interactions, advertising, and entertainment.

Volumetric displays render three-dimensional (3D) images directly onto a physical volumetric space^1^,^2^,^3^, enabling observers to view 3D images from any direction without the need to wear devices such as special glasses. This has motivated significant research efforts for the development and use of volumetric displays as a 3D visualisation technique. Moreover, an algorithm that can design 3D architectures of the volumetric displays exhibiting multiple two-dimensional (2D) images with directional characteristics has been proposed^4^,^5^.

Recently, volumetric displays that render 3D images in air using laser-induced plasma have been demonstrated^6^,^7^. Although these displays exhibit an important advantage in that aerial 3D images can be directly touched by a human hand, it is difficult to render a colour image in this approach because the colour of the plasma does not depend on the laser wavelength. Meanwhile, volumetric displays composed of fluorescent materials based on two-photon absorption have also been proposed^8^,^9^. These materials are excited at a focal point of an irradiated laser beam, and light emission is subsequently observed. Here, three primary-colour fluorescent materials (red, green and blue (RGB)) are employed for representing a full-colour image. Since the fluorescent materials of different colours must be placed at different positions relative to each other, these display devices require complicated structures, such as a stack of multiple thin layers.

In an alternative approach, Hashida *et al*. proposed a volumetric display based on photochromism^10^, which does not require the use of fluorescent materials. Photochromism is defined as the reversible phototransformation of a material’s structure between two forms with different absorption spectra^11^,^12^,^13^,^14^; the differences in the absorption spectra correspond to differences in the colour of the material. In a previous study, spiropyrans were used as the photochromic materials (PMs)^10^; this is a T-type PM that is coloured by ultraviolet light (UV) irradiation and is thermally decoloured when UV irradiation is blocked. To form volume elements (voxels), the T-type PM is coated onto transparent layered plates and a UV projector is used to individually control their forms (coloured/decoloured) to represent a 3D image. However, similar to other volumetric displays using a 2D projector^15^,^16^, a 3D PM array is severely restricted by the requirement to avoid the voxel overlap from the viewpoint of the projector. This prevents the use of high-density voxel arrangements and increases the size of the PM-based volumetric displays.

In this study, we demonstrate the underlying principle of a novel approach for full-colour volumetric displays based on thermally irreversible PMs (diarylethenes^11^,^12^), known as P-type PMs, and for the first time experimentally demonstrate the position- and colour-selective colourations of PMs. Most P-type PMs are transparent before being given a molecule-specific colour by UV irradiation. The decolouration of such a PM is only induced upon subsequent irradiation of the materials by visible (Vis) light with the appropriate spectrum. We exploit the intrinsic physical properties of P-type PMs with colour states that depend on both UV and Vis irradiations and develop novel design principles for 3D display applications.

Figure 1a illustrates the concept of the full-colour volumetric display based on P-type PMs. The volume space in which 3D images are rendered comprises a *mixture* of multiple PMs that assume different colours (cyan, magenta and yellow) upon UV irradiation. The colouration state of T-type PMs is determined by only one control light channel (UV). By contrast, for the P-type PMs used in this study, the colouration state depends on two control light channels (UV and Vis). As described below, by taking advantage of this feature, the volume where the 3D images are rendered can be constructed by uniformly mixing multiple PMs without considering the 3D alignment of the voxels.

We first describe the principle of *position-selective* PM colouration. We arrange the system such that the two control lights, i.e. UV light for colouration and Vis light for decolouration, are irradiated from different angles onto the PM volume (orthogonal in Fig. 1a). As a result of doing this, we detect a cross region where the UV and Vis lights intersect. When *both* UV and Vis lights are irradiated onto a PM, the PM exhibits *either* colouration or decolouration depending on the balance of the light power and absorbance of the materials. This phenomenon is based on the molecular properties associated with the potential energy surfaces (PESs), which determine the reaction paths of the colouration (open-ring to closed-ring) and decolouration (closed-ring to open-ring) reactions^13^,^14^. In the present study, we assume that a PM exhibits *decolouration* when *both* UV and Vis lights are irradiated. By spatially modulating the UV and Vis light patterns, their intersection can be arbitrarily and dynamically configured, achieving position-selective PM colouration.

Next, we present the principle of *colour-selective* colouration. When coloured by UV light irradiation, the three PMs (PM1, PM2 and PM3) in the system shown in Fig. 1 exhibit ideal absorption spectra of cyan, magenta and yellow, respectively (Fig. 1d). Consequently, PM1, PM2 and PM3 are decoloured by red, green and blue Vis lights, respectively (Fig. 1e). We now consider the case where both red Vis light and UV light are irradiated (Fig. 1b), considering that a PM device is a mixture of PM1, PM2 and PM3. In this case, only PM1 is decoloured; thus, the resultant colour of the overall PM is red. In the case where green Vis light and UV light are incident (Fig. 1c), only PM2 is decoloured and the resultant colour is green. In this manner, full-colour, colour-selective PM colouration is realised by controlling the Vis light spectrum (for decolouration). In other words, the principle of *colour-selective* colouration is based on spectrum-domain engineering of Vis light while exploiting the different PM-intrinsic colour attributes. Note that our system is constructed by merely mixing the multiple types of PMs; therefore, the structure of the display is extremely simple, unlike those of conventional displays, which require a designed 3D layout of nanomaterials. Furthermore, in this approach, the minimum voxel size is equal to the size of a molecule (~1 nm), enabling the development of miniaturized volumetric displays.

## Results

In this study, we experimentally demonstrate the principle of position selectivity using single-colour PMs. We use commercially available diarylethenes obtained from Yamada Chemical Co., Ltd. Figure 2a shows photographs of a PM dissolved in a toluene solution (coloured (right) and decoloured (left)). The transparent PM is coloured red by UV irradiation and the red PM is decoloured to transparency by Vis irradiation. Figure 2b shows the absorption spectra of the PM solution (Fig. 2a) before and after colouration, showing clear difference in the visible region. Figure 2c shows a photograph of the experimental setup. We form a transparent block that uniformly includes the PM by solidifying a mixture of the PM solution and a clear polydimethylsiloxane (PDMS) polymer (details are shown in the Methods section). The absorption spectra of the materials are barely affected by the inclusion in the polymer because PDMS is highly transparent in the UV–Vis region^17^. We refer to this block as the ‘PM volume’, which has dimensions of approximately 1 cm × 1 cm × 3 cm and is solidified to avoid the internal flow of coloured PMs. A UV source (peaked at 302 nm) is placed beneath the PM volume for uniform irradiation. As the Vis light source for the irradiation of arbitrary 2D patterns, we use a projector located to the left of the PM volume. As described above, decolouration is induced in the regions where both UV and Vis lights are incident and the PM volume is coloured only in the region that is irradiated solely by UV light.

**Figure 1:**
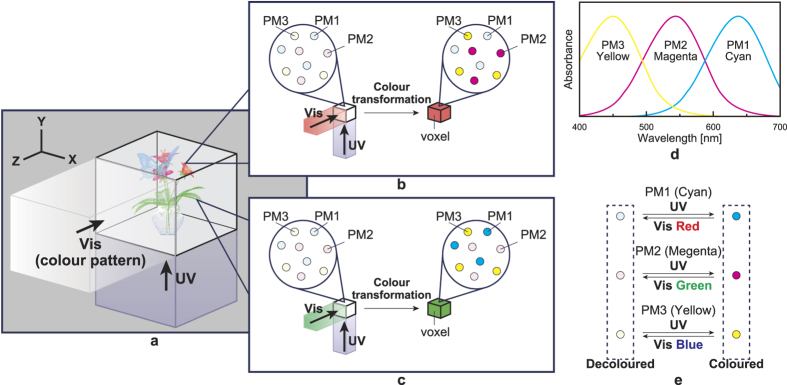
Concept and principles of the volumetric display based on a mixture of multi-colour photochromic materials. PM: photochromic material; UV: ultraviolet; Vis: visible. (**a**) Scheme of the proposed volumetric display. (**b**) Voxel colour transformation (Vis = red). (**c**) Voxel colour transformation (Vis = green). (**d**) Colour response of each PM.

**Figure 2:**
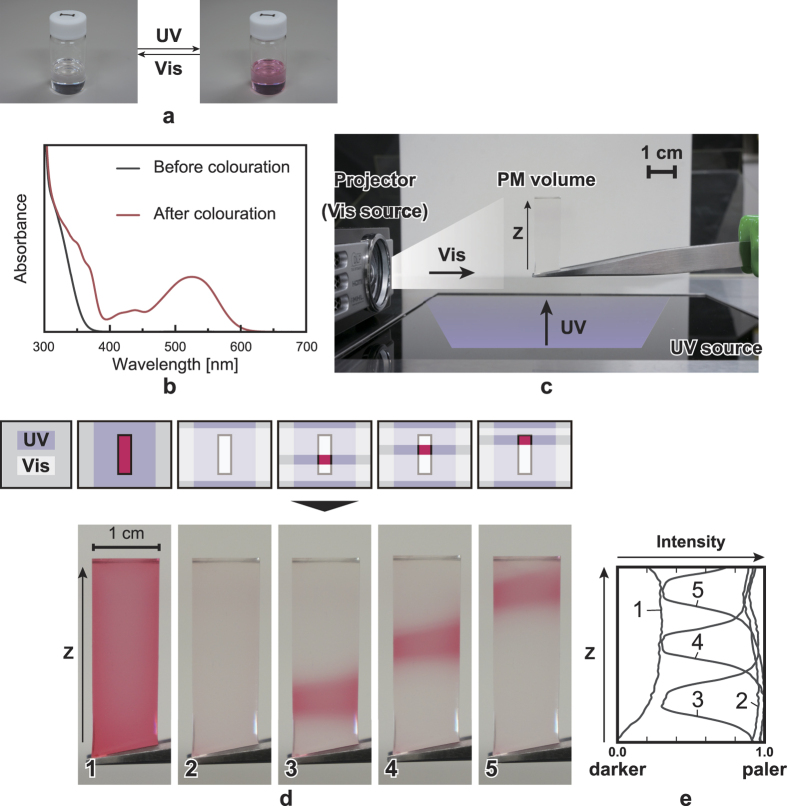
Fundamental experiment using a PM of one colour. (**a**) PM dissolved in toluene solution is coloured by UV irradiation and decoloured by Vis irradiation. (**b**) Absorption spectra of PM solution. For decolouration, PM must absorb light in the visible range. (**c**) Experimental setup. (**d**) Colour changes of PM volume after irradiations (bottom) performed in the illustrated manner (top). (**e**) Change in pixel values (intensities) of photographs in Fig. 2d with respect to depth direction (Z axis).

**Figure 3:**
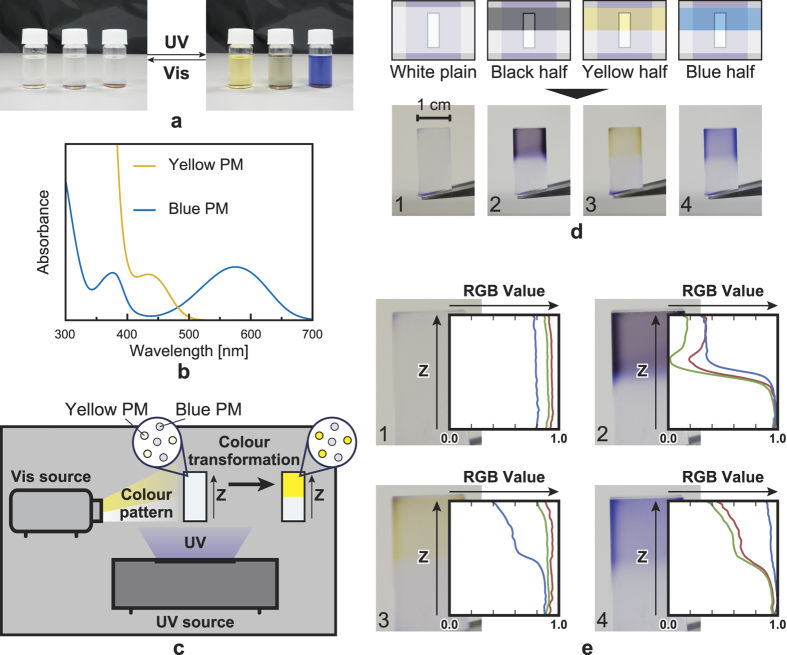
Experimental demonstration of position- and colour-selective colouration of a multi-colour PM mixture. (**a**) PMs dissolved in a toluene solution. Each solution includes, from left to right, yellow PM, both yellow and blue PMs and blue PM. (**b**) Absorption spectra of the coloured PM solutions (yellow and blue). Each PM requires a different light wavelength for the decolouration. (**c**) Experimental setup. (**d**) Colour change in the PM volume (bottom) after irradiation by UV or Vis light in the illustrated manner (top). (**e**) Change in the pixel’s RGB values for the images in Fig. 3d with respect to the depth direction (Z axis). The axes represent spatial position (vertical) and RGB values captured by a CMOS camera (horizontal).

Figure 2d shows photographs of the colour changes in the PM volume after light irradiation (lower) depending on the illumination of UV and Vis lights in the specified manner (upper). First, the entire PM volume is coloured red by UV irradiation (Fig. 2d-1). Then, this volume becomes transparent after simultaneous UV and Vis irradiations because of the dominance of decolouration by Vis irradiation (Fig. 2d-2).The UV and Vis light intensities are 0.84 mW/cm^2^ and 15.04 mW/cm^2^, respectively. Here, the projector illuminates a plain white pattern. Next, we induce position selectivity in the Vis pattern by altering the 2D pattern provided by the projector. As shown in Fig. 2d-3, the PM volume is coloured only in the regions where Vis light is not incident (i.e. the regions that are only irradiated by UV light). However, upon irradiation by UV light only, some of the regions that are not intended to and should not be coloured are, in fact, coloured as well. This can be explained as follows. Let us denote the arrival times and durations of UV and Vis irradiations by *T*_*UV*_, *T*_*Vis*_, *ΔT*_*UV*_ and *ΔT*_*Vis*_, respectively. If *T*_*UV*_ < *T*_*Vis*_, unintended coloration is present for the time period from *T*_*UV*_ to *T*_*Vis*_. Therefore, the arrival timing should be controlled carefully to avoid the arrival of UV before Vis. Next, consider the case of exactly controlled arrival timing (namely, *T*_*UV*_ = *T*_*Vis*_). If *ΔT*_*UV*_ > *ΔT*_*Vis*_, unintended coloration will be caused at the time period from *T*_*Vis*_ + *ΔT*_*Vis*_ to *T*_*UV*_ + *ΔT*_*UV*_. Thus, strict management of arrival timing *and* irradiation durations is required.

The curves in Fig. 2e show the normalised pixel values of the grey-scale images of the PM volume (Fig. 2d). Here, the Z axis represents the vertical direction (depth direction from the UV source). Higher values indicate more pale (or transparent) colour and lower values indicate darker colours. Examination of curves 3–5 confirms the successful realisation of the position-selective colour transformation principle.

In the experiments described above, the UV light source uniformly irradiates the volume, and therefore, the position selectivity of the PM colour transformation is confined to the depth direction (vertical axis in Fig. 2c). Nevertheless, rendering of an arbitrary 2D pattern for UV light is also possible by using appropriate modulation devices such as digital mirrors and galvanometer mirrors.

Next, we experimentally demonstrate the principle of representing multi-colour images. We use PMs of two colours: yellow and blue (Fig. 3a). The solution that includes both yellow and blue PMs is brown, as shown in the middle of Fig. 3a. Figure 3b shows the absorption spectra of the yellow and blue PMs after colouration by UV irradiation. The absorption bands of the spectra do not overlap in the visible range. Thus, colour-selective transformation is achieved by controlling the Vis irradiation spectra. Following the same procedure that was used to form the single-colour PM volume, we create a volume that includes both yellow and blue PMs. Here, the PM volume ha**s** dimensions of approximately 1 cm × 1 cm × 2 cm. Figure 3c shows the experimental setup.

**Figure 4:**
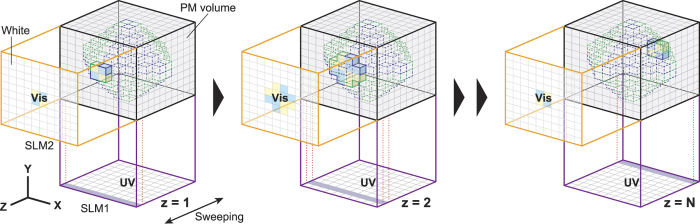
Volumetric display system based on the position- and colour-selective colour transformation method for PMs. SLM: space light modulator. UV and Vis irradiations are synchronously controlled by SLM1 and SLM2, respectively. SLM1 (UV) sweeps a 2D image rendered by SLM2 (Vis) along the depth direction (Z axis) to represent a 3D image on the volume. Voxels irradiated by white light or not irradiated by UV light would not be coloured, but transparent. Colour of each voxel is determined by the intensity ratio of the three primary colours in Vis light.

When the plain white Vis pattern illuminates the volume together with UV irradiation, the volume remains transparent because both yellow and blue PMs are decoloured (Fig. 3d-1). When only the lower half of the volume is illuminated by a white Vis pattern, the upper half of the volume turns dark brown because neither yellow nor blue PM is decoloured, as shown in Fig. 3d-2 (with the lower half remaining transparent because it is only irradiated by white light). When half of the Vis illumination pattern is yellow, only the blue PM is decoloured by the yellow light irradiation because the yellow PM does not absorb yellow light. As a result, the volume is coloured yellow, as shown in Fig. 3d-3. Figure 3d-4 shows the same effect for the case of the blue Vis illumination pattern.

The curves in Fig. 3e show the normalised RGB values of the pixels in the PM volume images (Fig. 3d). As in Fig. 2e, the Z axis in Fig. 3e corresponds to the vertical direction. The dark brown regions of the PM volume show low red, green and blue values (Fig. 3e-2), indicating that both yellow and blue PMs remain coloured in the volume. The yellow-coloured volume exhibits high red and green (red + green = yellow) values and low blue values (Fig. 3e-3). Similarly, the blue-coloured volume exhibits high blue values and low red and green values (Fig. 3e-4). These results confirm the successful realisation of the colour-selective transformation principle. Once again, we note that the PM volume is merely a uniform mixture of PMs with two different colours. This enables our demonstration that adequate UV and Vis light modulations can be used to provide an arbitrary colour at an arbitrary depth for the PM volume.

## Discussion

We now discuss the volumetric display system based on the principles described above (Fig. 4). The UV and Vis irradiations should be synchronously controlled by appropriate spatial light modulators (SLMs), denoted SLM1 and SLM2, respectively. Now, consider the case where the depth direction (*z** *****= **1, 2 … *N*) of a 3D image is controlled by SLM1. When only the region corresponding to *z** *****= **1 is irradiated by UV light from SLM1 and Vis light with a corresponding 2D pattern irradiates the region through SLM2, the 2D pattern is rendered onto the depth irradiated by UV light. Similarly, we can sequentially render arbitrary 2D patterns at *z** *****= **2 … *N*. Here, we assume that the rendering time of a 2D pattern at any depth is *Δt*. Therefore, it takes *NΔt* to scan through the thickness of the volume. If *NΔt* is shorter than the time resolution of human eyes, the observers recognize the sequential 2D patterns as a 3D image. The colour of each voxel is determined by its Vis light spectrum, corresponding to the configuration of the intensity ratios of the three primary colours (red, green and blue) in a conventional projector.

Finally, let us remark upon future research. We first address the frame rate of the display, and assume that to suppress flicker, the volume display frame rate must be at least 60 Hz (a common display frame rate). In the case where the depth resolution of the display is *N*, the 2D image rendering process should be completed within 1/(60 *N*) s at any depth. Since the colour change dynamics of a diarylethene molecule occur on the picosecond timescale^12^, the frame rate of the SLMs is the dominant bottleneck of our proposed system. Recently, various high-frame rate SLMs have been developed for display applications^18^,^19^. We believe that these devices can thoroughly cover the specifications of a future system based on the principles demonstrated in this study.

Our second remark concerns the display resolution. In theory, the depth resolution (Z axis) is dominated by the resolution of the images projected by SLM1, whereas the horizontal and vertical resolutions (X and Y axes) depend largely upon the resolution of the images shown by SLM2. Furthermore, the diffraction limit of light sets the size of the minimum light spot. However, experimentally, it was found that the boundaries between the coloured and decoloured areas are blurred on scales much larger than the light wavelength, as shown in the image in Fig. 2d as well as intensity profiles in Fig. 2e. Curves 3, 4 and 5 change gradually between the colouration and decolouration states. We attribute the blurring to an extra decolouration of the PMs by the spread of Vis light at the boundaries. The blurring was also observed in the multi-colour experiments (Fig. 3d-2 and 3e-2). The volume became blue at the boundary because only the yellow PM was decoloured by the spread of Vis (the yellow PM is decoloured more easily than the blue PM). This issue can be resolved using a highly directional light source such as a laser-based projector^20^.

In summary, we have proposed and experimentally demonstrated the position- and colour-selective colouration of PMs in a solid mixture of multiple PM types by modulating two control lights (UV and Vis). Spatial addressing was achieved by geometrical optical intersection of UV and Vis lights in the PM volume, while colour selectivity was based on the spectrum-dependent decolouration via Vis light; this is an intrinsic property of the PMs. By modulating the control lights, a dynamic and full-colour volumetric display was realised, meaning that arbitrary regions in the PM volume were configured to the designated colour. This paper presents a novel approach for the development of volumetric displays that can be used in the fields of information display and human–computer interaction, while demonstrating novel applications for PM materials in the fields of chemistry and material science.

## Methods

### Volume preparation

**Figure 5:**
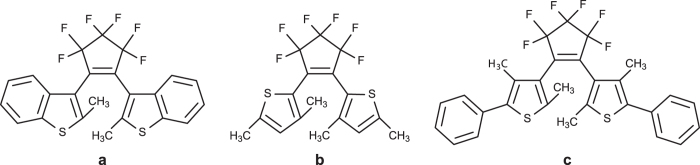
Chemical structures of (**a**) red PM, (**b**) yellow PM and (**c**) blue PM used in this study.

We used photochromic diarylethene molecules. Among many photochromic molecules, the diarylethenes derivatives that are used in this study are one of the most well-studied molecules due to their resistance to photo-fatigue. Their physical and chemical properties are well studied and have been documented in spectroscopy, thermal kinetics and photochemical kinetics investigations^13^. These compounds are commercially available and were purchased from Yamada Chemical Co., LTD. The catalogue numbers of the red, yellow and blue compounds are ‘DAE-0004’, ‘DAE-0068’ and ‘DAE-0018’, respectively. Their molecular formulas are given as C_23_H_14_F_6_S_2_ (red), C_17_H_14_F_6_S_2_ (yellow) and C_29_H_22_F_6_S_2_ (blue); their chemical structures (open-ring) are shown in Fig. 5. Here, all PMs have HPLC purities higher than 99%.

The PM volume used in the first experiment (Fig. 2) was composed of a PM that was coloured red upon UV irradiation. The PM was dissolved in a toluene solution (2 mg PM per 1 mL toluene), as shown in Fig. 2a. The volume was composed of a transparent polymer and the PM solution—with SYLGARD 184 Silicone Elastomer Kit (Dow Corning Toray Co.), which included two liquids (the base and the curing agent) and has been used in the literature^17^ as a polymer. The base liquid mostly consists of dimethyl siloxane dimethylvinyl-terminated and includes demethylvinylated and trimethylated silica. The curing agent is primarily made of dimethyl methylhydrogen siloxane trimethylsiloxy-terminated. The two liquids are solidified by mixing in a ratio of 10:1. The solidified polymer had a very high transparency in the UV–Vis region. The procedure for producing the PM volume is as follows:

1. We stirred 4.4 mL of polymer and 2 mL of PM solution.
2. We poured the mixture into a 1 cm × 1 cm × 4 cm mould and defoamed it under vacuum.
3. We heated the mixture at 100 °C for approximately 3 h to solidify.
4. We removed the solidified mixture from the mould and cut it to dimensions of 1 cm × 1 cm × 3 cm.

To enable the realisation of a multi-colour display, the PM volume used in the experiment (Fig. 3) was composed of yellow and blue PMs and was prepared in the same manner as described above. The two PMs were dissolved in a toluene solution (1 mg yellow PM and 1 mg blue PM per 2 mL toluene), as shown in the centre of Fig. 3a. The volume was fabricated by mixing 4.4 mL of polymer and 2 mL of the PM solution.

### Equipment for the experiment

A 302-nm-wavelength UV source (AS ONE, ‘MID-170’, Transilluminator) was used in the experiments and a projector (Vivitek, ‘QUMI Q5’) was used as the Vis source. The specifications of the projector are as follows.

- Brightness, 500 ANSI Lumens.
- Native resolution, 1280 × 800 pixels.
- Contrast ratio, 30,000:1.

### Quantification of the results

The curves in Fig. 2e were drawn from the photographs in Fig. 2d. The intensities in Fig. 2e indicate the average pixel values of the grey-scale photographs. The average pixel values were calculated over an area of 32 × 32 pixels. Here, the depth direction from the UV source was defined as the Z axis. The curves in Fig. 3e were drawn from the photographs in Fig. 3d in the same manner but were not grey-scaled.

## Additional Information

**How to cite this article**: Hirayama, R. *et al*. Optical Addressing of Multi-Colour Photochromic Material Mixture for Volumetric Display. *Sci. Rep.* **6**, 31543; doi: 10.1038/srep31543 (2016).
